# An Experimental Strategy for Characterizing Inductive Electromagnetic Energy Harvesters

**DOI:** 10.3390/s20030647

**Published:** 2020-01-23

**Authors:** Pedro Martín Sánchez, Fco. Javier Rodríguez Sánchez, Enrique Santiso Gómez

**Affiliations:** Department of Electronics, University of Alcalá, Alcalá de Henares, 28805 Madrid, Spain; pedro.martin@uah.es (P.M.S.); Enrique.santiso@uah.es (E.S.G.)

**Keywords:** energy harvesting, condition monitoring, inductive harvesting

## Abstract

Condition monitoring of high voltage power lines through self-powered sensor systems has become a priority for utilities with the aim of detecting potential problems, enhancing reliability of the power transmission and distribution networks and mitigating the adverse impact of faults. Energy harvesting from the magnetic field generated by the alternating current flowing through high voltage lines can supply the monitoring systems with the required power to operate without relying on hard-wiring or battery-based approaches. However, developing an energy harvester, which scavenges the power from such a limited source of energy, requires detailed design considerations, which may not result in a technically and economically optimal solution. This paper presents an innovative simulation-based strategy to characterize an inductive electromagnetic energy harvester and the power conditioning system. Performance requirements in terms of the harvested power and output voltage range, or level of magnetic core saturation can be imposed. Different harvester configurations, which satisfy the requirements, have been produced by the simulation models. The accuracy and efficiency of this approach is verified with an experimental setup based on an energy harvester, which consists of a Si-steel magnetic core and a power conditioning unit. For the worst-case scenario with a primary current of 5 A, the maximum power extracted by the harvester can be as close as 165 mW, resulting in a power density of 2.79 mW/cm^3^.

## 1. Introduction

Condition monitoring of high-voltage power lines plays an important role in the design and operation of electrical power networks, providing a comprehensive view of the state of the transmission power systems or the electric grid infrastructure. The purpose of condition monitoring is twofold: Firstly, it boosts revenue by reducing installation, maintenance and operating costs. This is especially true when self-powered and autonomous sensors are used, which allow the utility shutdown to be reduced, since battery-powered sensors have a fixed and limited lifespan. Secondly, condition monitoring improves supply reliability since periodic inspections are required, although these are usually carried out by less reliable, traditional, on-ground visual means. The significant advantages condition monitoring brings to the grid operators, can however be outweighed by the operating costs and maintenance requirements of monitoring sensors. Needless to say, this runs counter to the reduction of the reinforcement and maintenance costs grid operators are seeking to achieve these days. Finally, condition monitoring allows predictive rather than corrective maintenance to be done, prolonging the useful life of the assets in the grid at the expense of increased risk of failure. This risk can be mitigated by collecting accurate and real-time information about the performance and operating condition of the grid. 

Energy harvesting as power source for condition monitoring is increasingly being investigated as an attractive alternative to batteries, particularly in low power sensing applications. Likewise, the advances in wireless network technologies and the reduction in power requirements of electronic devices have paved the way to independent remote sensing nodes. These features have made condition monitoring a viable reality.

**Figure 1:**
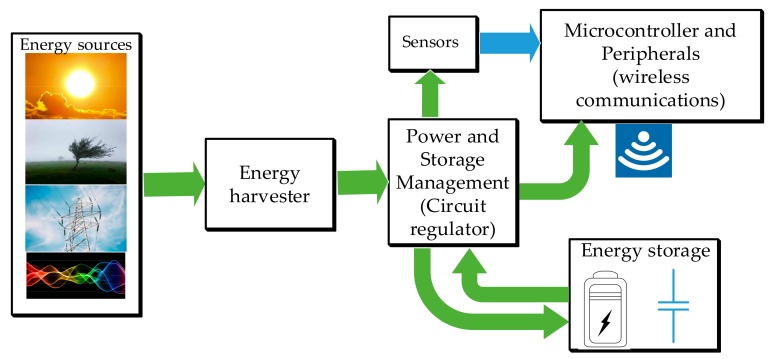
Block diagram of an energy harvesting system.

In the literature, there is a broad range of different architectures for energy harvesting systems. However, all of them have some blocks in common as depicted in Figure 1: (1) the energy harvester; (2) the energy conditioner or power conditioning circuits; (3) the energy storage; and (4) the system to be powered, which usually includes a microcontroller, sensors and communication peripherals, among other components. 

Regarding the energy sources and harvester, there are several alternatives that have been reported in the literature. Wind power [1,2,3,4], solar energy [5,6] or hybrid wind/solar approaches [7] are used for harvesting power not only in the range of μW and mW but also in medium power range (1–10 W) [7]. The energy harvester can be an anemometer [1], flexible piezoelectric devices [2] or small scale wind turbines with an electrostatic converter [3,4] for the wind-based alternative, or solar panels for solar-based energy harvesting [6] (pp. 1045–1046). Solar panels require, however, regular maintenance, such as cleaning when they are covered with dust, snow or ice, since maximum performance is desired. The mechanical energy of vibration can also be turned into electricity by using one of following three approaches [8]: piezoelectric, electromagnetic, often modeled as an inductive spring mass system [9] or electrostatic. The energy harvested from vibration can be improved by implementing several strategies. For example, in [10] the authors describe an approach of a linear electromagnetic vibration energy harvester with weak magnetic coupling in which the energy harvested is enhanced by implementing the energy localization phenomenon. Likewise, a hybrid piezoelectric-electromagnetic vibration energy harvester is introduced in [11]. In order to improve performance of the vibration energy harvester, in [12] an approach based on an array of coupled levitated magnets is proposed. This approach, which is based on the single levitated magnet design proposed in [13], combines the benefits of nonlinearities and modal interactions. The use of nonlinearity to boost performance is also considered in [14] where a nonlinear vibration energy harvester based on the concept of high static low dynamic stiffness is proposed. In [15] with the aim of broadening the bandwidth of the effective harvesting frequency, a multi-frequency energy harvester array consisting of three permanent magnets, three sets of two-layer copper coils and a supported beam, is introduced. Broadening the bandwidth is also a primary objective accomplished in the work presented in [16]. By using a series of cantilevers with different lengths and resonance frequencies, they manage to widen the overall bandwidth, as well as to increase the generated power. In general, ambient vibrations are random and the vibration spectra vary enormously for different applications, which makes the design of vibration-based harvesters a challenging task. Energy contents in heat can also be scavenged by using thermoelectric generators, which obtain the energy in the form of electricity when there is a difference in temperature -Peltier-Seebeck effect-[17]. For instance, a thermoelectric generator can be used for human body heat energy harvesting with the aim of powering wearable devices [18,19]. Radio frequency-based harvesting approaches obtain the energy from radio waves. According to the RF energy source being considered, there are two categories [20,21]: (i) RF signal from dedicated sources; and (ii) RF energy from ambient sources, such as telecommunication towers and mobile devices, in which wireless communication devices can act as RF sources to power their neighbouring devices. The low power conversion efficiency of RF harvesting and the ultra-low voltages that are incident at the receiver antenna constitute a formidable challenge that can limit its applicability [22]. Electric field energy harvesting is another alternative presented in many papers in literature [23,24,25,26,27] based on the principle that an energized conductor creates a radial electric field. The advantage of this technique is that the energy can be harvested from a no-load AC power line without current flowing through it. The electric field is always present around the high voltage power lines and it is stable and predictable since the voltage is regulated. In general, the energy is obtained from the electric fields around the power lines by using the principle of a capacitor divider. However, for the same amount of power, the size of the electric-field-based harvester must be greater than that of the magnetic-field-based alternative. The energy density obtained from a magnetic field is considerably higher compared to an electric field [23]. In addition, the high voltage involved with electric fields, challenges the technical feasibility of its implementation, increasing the associated cost: the installation of this type of harvesters requires a major power shutdown since they are usually either attached under the line or coiled around the cable [25]. The energy harvesting from the magnetic fields surrounding high-voltage lines has received considerable attention in recent years. There are two approaches as a function of the way the harvester is located [28,29,30,31,32,33]: (i) free-standing when the harvester is located in close proximity to the conductor without enclosing it; and (ii) the current-transformer-based inductive harvester which encloses the conductor to obtain maximum magnetic coupling [28]. Current transformers (CTs) are generally used to measure AC amperage by sensing the magnetic field generated by the primary current flowing in the conductor around which the CT is installed. However, CTs can also be used for inductive energy harvesting. The performance of the inductive harvester greatly depends on the magnetic core material characteristics, such as the magnetization curve, permeability, power losses and saturation [29]. Although saturation is nearly always avoided, in [31] the authors revealed that for any given core, regardless of the application, power harvest is maximized when the core is permitted to saturate at a particular time window within the line cycle. An alternative approach is presented in [30], where a miniaturized linear permanent magnet synchronous generator is utilized. The magnetic field is first converted into mechanical vibration by using a permanent magnet and then the vibration energy is turned into electricity by using a synchronous generator. 

As far as the power and storage management circuit are concerned, power electronics are required for several reasons. Firstly, high power extraction involves impedance matching between the energy harvester, and the load, i.e., when the load impedance equals the complex conjugate of the source impedance [28,29,32,33,34,35]. Secondly, some form of voltage regulation is essential owing to the fact that the output current and voltage generated by the harvester do not usually match with the load requirements. Finally, the intermittency of the energy source should not affect the continuous operation of the system. Therefore, some form of energy storage is generally included. In [34] the impedance matching is achieved by a compensating capacitor along with a buck-boost converter which assure constant load impedance. The energy is stored in a rechargeable battery. In [36] the authors introduced an adaptive approach, which achieves automatic power optimization by using a dc-dc converter thereby maximizing the power stored in a battery. Other works have used dc-dc converters to achieve maximum power transfer [37,38,39]. Batteries are the most frequent storage strategy for energy harvesting systems [5,34,36], Supercapacitors have also been used to buffer energy so that the system can operate in a power outage [7,40,41,42,43]. The physical deterioration of the rechargeable batteries constitutes the limiting factor of the lifetime of the energy harvester. The premature aging of the cells occurs when the battery is subjected to repeated charge/discharge cycles. In order to prolong the lifetime of the battery, in [44] is presented an implementation that uses a two-stage storage system consisting of supercapacitors and a lithium rechargeable battery.

This paper presents a simulation-based strategy for characterizing a CT-based inductive electromagnetic energy harvesting system, in terms of the core material through its magnetization curve, number of turns in the secondary winding, magnetic path length and cross-sectional area of the core, copper cross section of the winding, primary current, length of the copper wire, load resistance and compensating capacitor, inter alia. This work introduces a new energy harvester design strategy aiming at extracting the maximum power from a high voltage line. An experimental setup with different configurations validates this strategy. The MATLAB/Simulink tool is used for modeling the energy harvesting system and for data visualization. The system is able to accurately estimate the matching impedance, the optimal dimensions of the coil and the power harvested for different primary current conditions and different loads. As a result, a rapid prototype design is developed. 

The remainder of this paper is organized as follows. In Section 2 the CT-based energy harvesting system model is introduced. The linear and non-linear behavior of the model is analyzed and the Simulink-based electrical model is defined. By using an electrical model, Section 3 deals with the simulation-based characterization of an inductive electromagnetic energy harvester. Section 4 introduces the prototype of the harvester used for model validation purposes. Finally, some conclusions are drawn in Section 5.

## 2. CT-based Energy Harvesting System Model

In this section, an electrical circuit model of the inductive electromagnetic harvester based on the electromagnetic theory and the equivalent circuit of a current transformer is presented. As stated in the introductory section, CTs are generally used to measure line currents in electric power systems. This is done by sensing the magnetic field generated by the current. In that context, the main purpose of CT is to translate the primary current in a high voltage power line into the secondary current whose value is directly proportional to the primary current and inversely proportional to the number of turns in the secondary winding. However, current transformers can also be used as energy harvesters to power electronics attached to the power line for condition monitoring purposes. In fact, a CT is a preferred device to harvest power in the grid nowadays. There are several reasons for this assumption. Firstly, the current-transformer-based inductive harvester encloses the conductor to obtain maximum magnetic coupling. As a result, the power density of this type of energy harvester is relatively high and maximum amount of power can be extracted from the magnetic field. Secondly, the installation of this type of CT-based harvesters does not require a major power shutdown, provided a split magnetic core is used. Thirdly, the CT is galvanically isolated from the high voltage power line. Therefore, in principle a malfunction of the CT does not degrade the reliability of the monitored system. Finally, the CT tolerates harsh operating conditions without degradation in performance. 

### 2.1. Equivalent Electrical Circuit of the Energy Harvester

**Figure 2:**
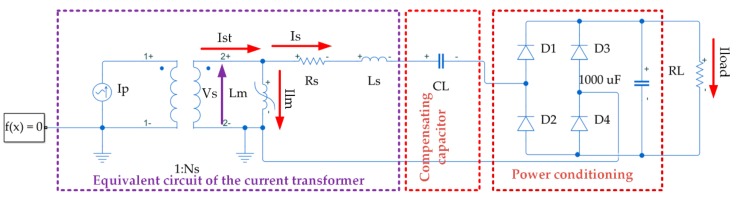
Equivalent electrical circuit of a CT-based electromagnetic energy harvester.

Figure 2 shows the equivalent circuit of a current transformer along with rectified-based power conforming circuitry and compensating capacitor. This MATLAB/Simulink-based model is used to simulate the performance of a CT as an energy harvester. The CT mainly consists of an ideal transformer and a nonlinear inductor. The primary winding is energized with an alternating current with peak value I_p_, which generates the magnetic field, H, and the flux density B inside the core. This induces a voltage in the secondary winding and, when a resistive load is connected to the harvester, the current flowing through it is proportional to the primary current, provided the core is not in hard saturation. 

In Figure 2, R_S_, the secondary leakage resistance, represents the power loss of the secondary winding. The magnetic flux losses of secondary windings are represented by the leakage inductance Ls. If the magnetic core has high permeability, most of the mutual linkage flux is confined to the core. The leakage flux can be assumed proportional to the current producing it and it depends on the geometry of the winding and core. Therefore, it can be assumed that the leakage inductance, Ls, is constant accounting for the voltage drop induced by the leakage flux. Finally, C_L_ is used to compensate the inductive behaviour of the harvester aiming to harvest the maximum power available at low primary current, the startup primary current, at which the available power is limited. 

### 2.2. Linear and Nonlinear Behavior of the Energy Harvester Model. 

Considering that the harvester is based on an ideal CT, the current flowing through the secondary winding can be evaluated by using the amp turn equation:

(1) $$N_{p}I_{p}\;=\;N_{s}I_{st}$$

where N_S_ is the number of turns in the secondary winding and N_P_ = 1 for the CT in Figure 2. Hence, the maximum total secondary current, I_st_, can be expressed as:

(2) $$I_{st}=I_{s}+I_{lm\;}\;=\;\frac{I_{p}}{N_{s}}$$

where I_lm_ is the magnetizing current, whose instantaneous value can be written as:

(3) $$i_{lm}\left(t\right)\;=\;\frac{1}{L_{m}}\int v_{s}\left(t\right)dt$$

**Figure 3:**
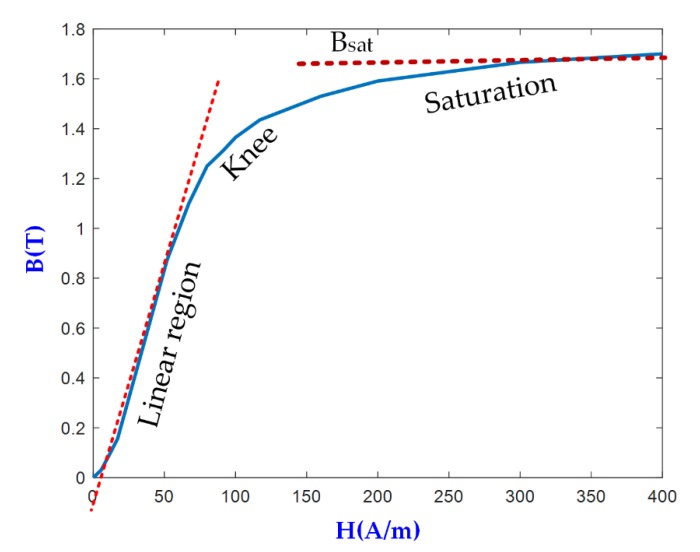
Magnetization curve of the non–linear inductor.

While the magnetic core is not saturated, i.e., I_lm_ ≈ 0, the harvested current going to the load, I_s_, is only determined by the primary current divided by N_S_. The magnetic core, however, exhibits nonlinear behavior, which in Figure 2 is represented by L_m_, a nonlinear inductor that plays the role of the CT magnetization inductance. L_m_ can be modelled by using the magnetization curve of the core, i.e., the magnetic flux density versus magnetic field strength characteristic, the core cross sectional area,$\;A_{eff}$, the magnetic core path length, $l_{eff},\;$and N_s_. These parameters are used in the Simscape model of the non-linear inductor, which can be specified with varying levels of nonlinearity [45]. Figure 3 shows the magnetization curve, also called B-H curve, of the nonlinear inductor in Figure 2. H and B correspond with the magnetic field strength and the magnetic flux density, respectively. The nonlinear behaviour of the CT is represented by the B-H curve, which in turn constitutes the magnetization curve of the core. In the linear region of the magnetization curve when the relative permeability is constant, L_m_ can be express by using Equation (4):

(4) $$L_{m}\;=\;\frac{\mu_{0}\mu_{r}A_{eff}N_{s}^{2}}{l_{eff}}$$

where $\mu_{0}$ is the permeability of free space, i.e., 4π x 10^−7^ H/m, and $\mu_{r}$ is the relative permeability, which depends on the core material and can be estimated from the magnetization curve (see Figure 3). The specific values for the rest of parameters are given in Section 4 for the proposed harvester in this paper. 

For a core material with high permeability, the magnetizing inductance takes a high value and the harvester works as an ideal CT. This is the ideal CT operation that takes place in the linear region of the B-H curve where the core is not saturated, and the entire transformer current is delivered to the load. This is also the working region for a CT-based primary current measurement device, where the primary current can be estimated by measuring the secondary current, since they are proportional to each other. When the core is operating in the knee or in the flat tail regions in the B-H curve, the core is said to be saturated. Uncontrolled core saturation can bring about detrimental effects as stated below.

According to Faraday’s law, the voltage developed by the core is proportional to the time derivative of B. A sinusoidal AC current flowing through the primary conductor generates a time-varying magnetic field around the wire, and through the magnetic core the magnetic field is turned into a time-varying magnetic flux density (B(t)). Voltage is induced across the terminals of the secondary winding when the magnetic flux (Φ(t)) crosses the loops in the secondary winding. If B(t) is uniform over and perpendicular to the area A_eff_ of the coil, the voltage induced in a Ns-turn winding can be expressed by:

(5) $$v_{s}\left(t\right)\;=\;N_{s}\frac{d\emptyset \left(t\right)}{dt}\;=\;N_{s}A_{eff}\frac{dB\left(t\right)}{dt}\;=\;N_{s}A_{eff}\frac{d}{dt}\left(\frac{\mu_{0}\mu_{r}i_{p}\left(t\right)}{2\pi r}\right)\;=\;\frac{\mu_{0}\mu_{r}N_{s}A_{eff}}{2\pi r}\frac{d\left(i_{p}\left(t\right)\right)}{dt}$$

where *i_p_(t) = I_p_cos(ωt + φ)* is the sinusoidal AC current through the primary conductor and 2πr is the circumference of the circle in which the magnetic field is calculated. This value can be considered the magnetic path length, l_eff_, for a toroidal core. Then, Equation (5) can be expressed as:

(6) $$v_{s}\left(t\right)\;=\;\frac{L_{m}}{N_{s}}\omega I_{p}\sin\left(\omega t+\varphi \right)\;=\;L_{m}\omega I_{st}\sin\left(\omega t+\varphi \right)$$

From Equation (5), B(t) can be derived as follows:

(7) $$B\left(t\right)\;=\;\frac{1}{N_{s}A_{eff}}\int v_{s}\left(t\right)dt$$

where $\int v_{s}\left(t\right)dt$ is the applied volt-seconds. Saturation is caused by excessive applied volt-seconds, which places constraints on the magnetic flux density. This imposes limitations on the voltage applied to the nonlinear inductor, which clearly affects the allowed load voltage. In equation 7, it can be seen that saturation can also be prevented by increasing the core cross-sectional area or the number of turns in the secondary. 

One of the negative effects of core saturation occurs when the operating point in the B-H curve of the core is in the flat tail regions where B takes a constant value regardless of the variation in H and time (see Figure 3). As a result, the voltage induced across the terminals of the secondary winding is close to zero. In this case, virtually no current would be delivered to the load. In other words, when the core is operating in saturation, there is no energy harvested and power is only transferred into the load for a portion of the periodic cycle where the core is not saturated. The period of time when the core is able to transfer power can be referred as transfer window [31]. In [31] authors prove that for any given magnetic core, power extracted is maximized when the core is allowed to go into saturation in a part of the periodic cycle. The level of core saturation is not fixed at one operating point during harvesting operation since the core goes in and out of magnetic saturation thereby covering the entire unsaturated regions of the B-H curve along the way. Another negative effect of a saturated core is related to the evaluation of the capacitor used to compensate the inductive impedance inserted by the harvester. The maximum power extracted in the transfer window occurs if the load impedance is the complex conjugate of the source impedance. However, a direct impedance compensation is sometimes unfeasible for inductive electromagnetic energy harvesters, where a limited range of voltage values is required by the dc-dc regulator, which may impose the value of the load resistance. To compensate for the inductive impedance of the harvester, a series capacitor is connected at the output of the harvester (see Figure 2). Under core saturation, the magnetization inductance inevitably varies with time, thereby making it difficult to calculate the compensating capacitor. Finally, for a split core, the magnetostriction effect in saturation will cause noise and physical vibration, which will result in installation damage and performance deterioration. Clearly, core saturation significantly complicates an analytical model that could be used to guide the process of core characterization for maximum power extraction. 

In this paper a MATLAB/Simulink-based simulation model is implemented to characterize the energy harvester in terms of several parameters: (i) the minimum power required by the monitoring system; (ii) the load resistance to keep the core operating within the transfer window for the designed voltage range; (iii) the compensating capacitor to extract maximum power when the startup primary current flows through the primary conductor; (iv) the number of turns in the secondary; and (v) the maximum level of core saturation. This latter parameter cannot be obtained directly from the model. Two related parameters can be considered and measured to estimate the level of saturation, namely: (i) the Total Harmonic Distortion (THD) of the induced voltage in the secondary winding or the secondary current; and (ii) the deviation of the current in the secondary winding from the ideal value based on the current source behavior, i.e., value I_m_ in Figure 2. The benefits of letting the core go into soft saturation should outweigh the negative impact of saturation. Hence, the operating range of the core should not be beyond the onset of the knee region in Figure 3. This value is calculated by limiting the level of THD for the secondary current to a particular value, critical THD, which will depend on the harvester configuration. 

### 2.3. Saturation Characterization

**Figure 4:**
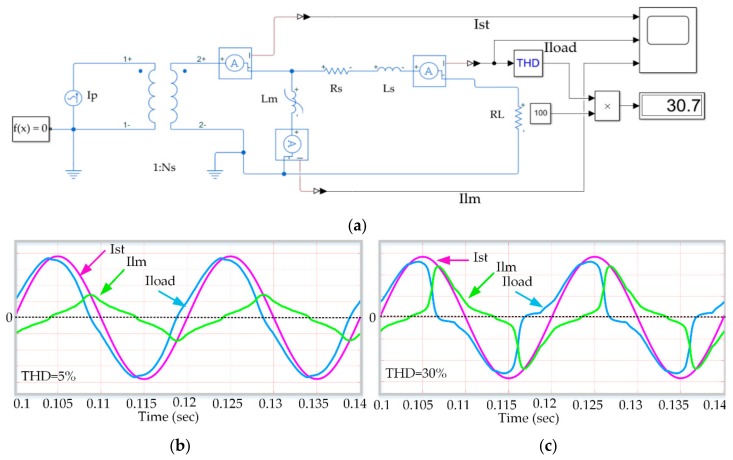
(**a**) Experimental setup for saturation characterization. (**b,c**) Saturated waveforms.

Figure 4 shows the experimental setup used to explore the relationship between the THD and the magnetic core saturation. The waveforms for currents involved are also depicted. For this experimental setup, the THD has been calculated for the load current.

When the magnetic core is not saturated or in soft saturation (Figure 4b) the magnetizing current, I_lm_ lags 90º the total secondary current, I_st_, on account of the resistive load connected for the harvester. In soft saturation, the time-varying value of the magnetizing inductance makes I_lm_ different from zero, which causes the distortion of the load current. By calculating the THD of the load current, the level of core saturation can be estimated. When the core goes through the knee region in the magnetizing curve towards deeper saturation, still without reaching hard saturation where B(t) = Bsat and the inductive voltage equals zero, I_load_ is virtually zero during a period of time within the line cycle. This leads to more distortion of I_load_, as can be seen in Figure 4c. Consequently, the THD value of I_load_, can be taken as an indicator of core saturation. This value will be used as an additional requirement to determine the load resistance and the compensating capacitor values for maximum power extraction purposes. 

## 3. Simulation-Based Characterization of the Inductive Electromagnetic Energy Harvester 

As stated above, the process of characterization of an inductive electromagnetic energy harvester is not straightforward, on account of the number of variables involved. In Section 2, these variables have been introduced: THD as an indicator of the saturation level, primary current (I_p_), number of turns in the secondary winding (N_S_), load resistance (R_L_), compensating capacitor (C_L_), core cross-sectional area, core magnetic path length and the core magnetization curve, inter alia. The key aim consists in defining the relationship between the output power and the aforementioned variables. In literature, the relationship among some of them has been determined through analytical models. However, these analytical models have to be combined with the power management circuits required for power conforming and voltage regulation. In this section, this relationship is defined by implementing a simulation approach based on the circuit depicted in Figure 2. To gain a clear insight about the influence of the compensating capacitor, two models are used: Model 1, which includes the resistive load and rectifier; and Model 2 with a resistive load, rectifier and compensating capacitor. Figure 3 represents the B-H curve of the non-linear inductor used in both models and the cross-sectional area A_eff_ and the magnetic path length l_eff_ correspond to the values of the core used in the prototype of the harvester shown in Table 3. It is important to remember that the energy harvester is modeled by an ideal transformer, the nonlinear inductor, and the secondary leakage resistance and inductance. 

### 3.1. Model 1. Simulation with an Energy Harvester with Rectifier and Resistive Load Without Reactive Power Compensation. 

This model is used to evaluate the amount of power that can be extracted by a harvester with rectifier and resistive load (see Figure 2). The purpose of this model is twofold: (i) to find out the minimum value of the primary current for a particular power value, the startup primary current, so that the harvester can scavenge the rated power for the monitoring system; (ii) to evaluate the range of load resistance values which make the harvester operate either within the linear region or in the knee region of the magnetization curve. 

**Figure 5:**
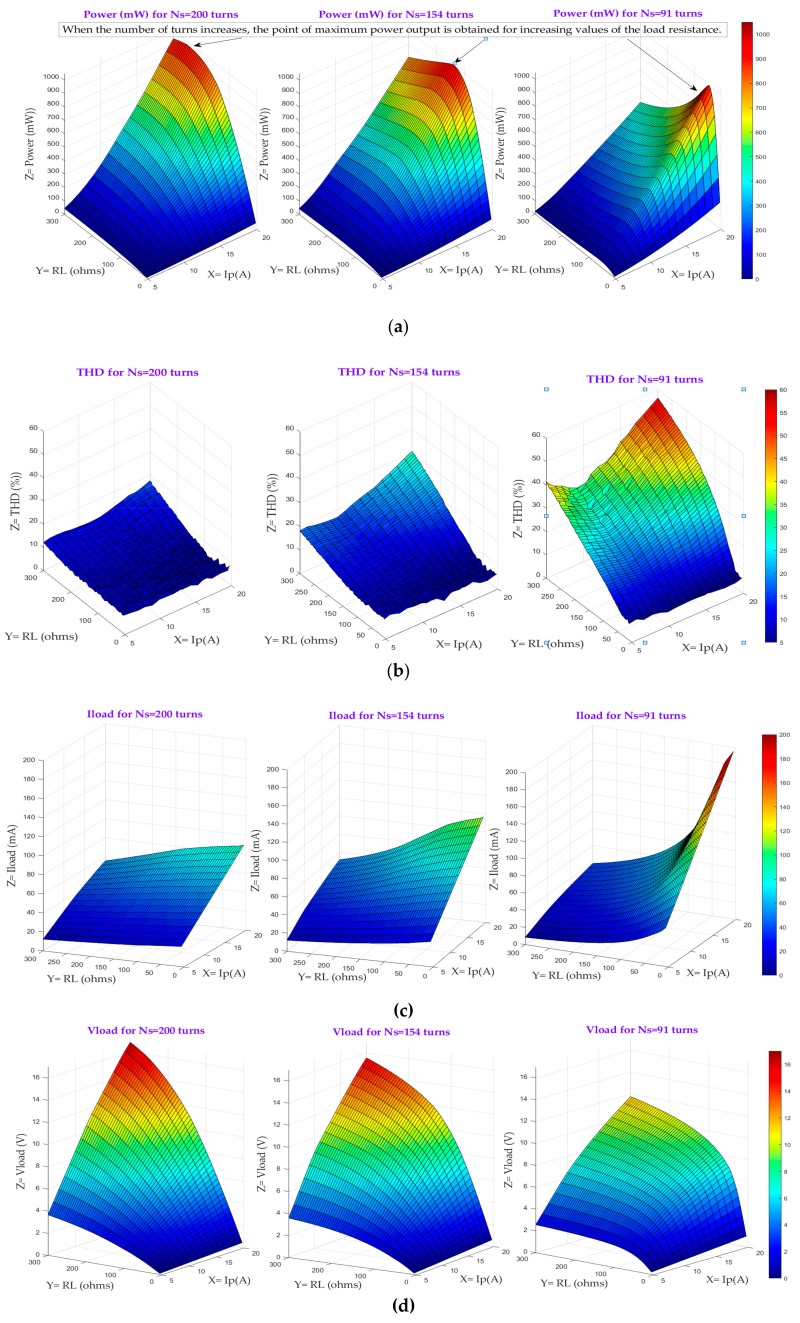
(**a**) Output power; (**b**) THD; (**c**) V_load_; and (**d**) I_load_ as a function of I_p_, R_L_ and Ns.

Figure 5 shows the output power (Figure 5a), the THD of the secondary current, Is, (Figure 5b), the load current (Figure 5c) and the load voltage (Figure 5d) as a function of the primary current, ranging from 5 A to 20 A at a step size of 1 A, the load resistance which takes values from 2 Ω to 200 Ω in 1–Ω steps and the number of turns in the secondary, Ns. 

From the Figure 5 some conclusions can be drawn. As far as the output power is concerned, when the core is at the onset of saturation, e.g. the THD is less than 5 %, the output power is directly proportional to the quadratic value of the primary current, and the harvested current is only determined by the primary current. This output power has been worked out by multiplying the rectified load current and the rectified load voltage. The same applies for the load resistance: the larger the load resistance the greater the power extracted, provided the THD is below a certain value, which depends on the number of turns of the secondary winding. When the number of turns increases, the point of maximum power output is obtained for increasing values of the load resistance. In other words, saturation is achieved earlier as the number of turns decreases (see Figure 5b). This is to be expected because the magnetic flux density, B(t), is inversely proportional to the number of turns and directly proportional to the volt-seconds accumulated in the core as stated in equation 7. The volt-seconds allowed by the core before entering into saturation are a function of saturation flux density level, Bsat, the effective cross sectional area, Aeff and Ns: the fewer the number of turns, the sooner Bsat is reached for the same amount of volt-seconds. As for the load current (Figure 5c), when the core is operating in the linear region, the current delivered to the load increases with decreasing number of turns. However, when the core goes into saturation, the magnetizing current increases thereby reducing the current delivered to the load. Finally, Figure 5d shows that the load voltage is linearly increased under non-saturation conditions for increasing value of the load resistance. In this scenario, the harvester is working as a current source, which is able to develop any arbitrary voltage required to sustain the current into the load. Conversely, when the core enters into saturation, the load voltage increase is no longer linear due to the fact that some of the harvested current is lost in the magnetizing inductance. 

Although Figure 5 shows a valuable insight into the complex relationship among the different variables, finding the right values for these variables, which characterize the harvester in order to comply with the system requirements, needs a more comprehensive analysis of the data generated in the simulation step. The value, or range of values, of these parameters will depend on the application and/or the system to be powered. The number of different possibilities makes the harvester characterization an arduous and time-consuming process.

To prove the effectiveness of the strategy presented in this paper, a set of requirements is established and the harvester configurations which satisfy the requirements are depicted in more easily readable form. Table 1 shows the possible harvester configurations. It has been assumed, that the power required for the system ranges from 150 mW to 600 mW, the primary current takes values between 5 A and 15 A, the voltage window at the input of the system ranges from 1 V to 3.3 V, e.g. a dc-dc regulator could place this limit, the THD should be below 7% to allow the core to enter the onset of the knee region or soft saturation, and for Ns = 200 turns

From Table 1 one definite conclusion can be reached: the minimum required power cannot be obtained with the startup primary current of 5 A. The same conclusion can be drawn for Ns = 154 and 91 turns. As a result, reactive power compensation should be included.

### 3.2. Model 2. Simulation with an Energy Harvester with Rectifier and Reactive Power Compensation 

**Figure 6:**
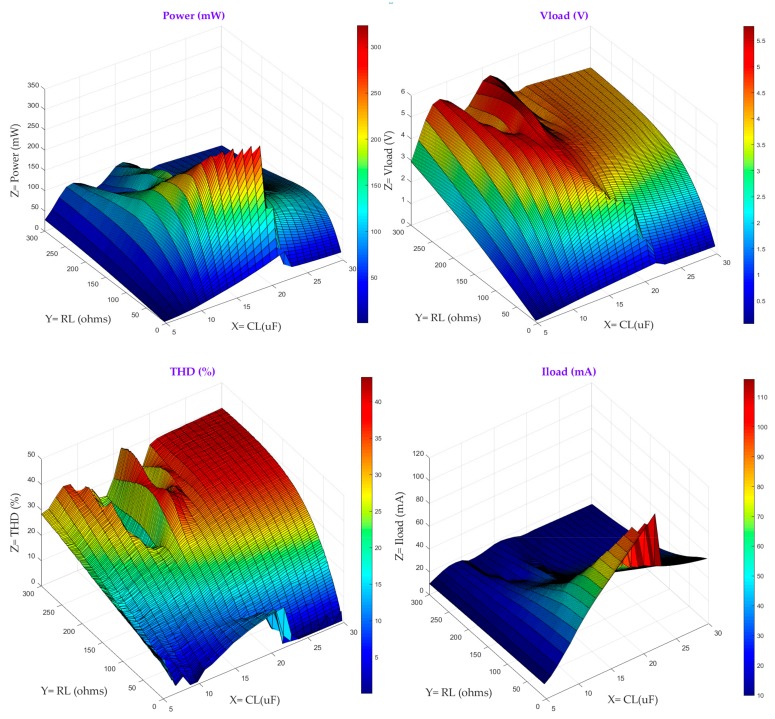
Power, THD, V_load_ and I_load_ as a function of R_L_ and CL for Ip = 5 A and Ns = 200.

Model 2 allows the compensating capacitor to be evaluated for maximum power transfer. When there is reactive power compensation, the energy harvester will provide more power to the load. This produces an undesirable side-effect on the harvester: higher power will increase voltage in the magnetizing inductance thereby increasing the magnetizing current and leading the core into saturation with smaller primary current. This fact can be observed in Figure 6, which represents the output power, the load voltage, the THD and the load current as a function of the load resistance and the compensating capacitor, C_L,_ with values ranging from 5 to 25 μF. For the simulation, Ns equals 200 turns, with the primary current being 5 A, since it is the worst case scenario. 

Figure 6 gives a basic insight into the relationships among the different variables considered for the harvester characterization and allows their values to be narrowed down. For instance, for values of CL greater than 20 uF the output power plummets since there is no impedance matching. As for the resistive load, for values greater than 100 Ω, the THD values clearly indicate that the magnetic core is likely to be saturated, although there is a region in the THD curve with low level of THD for values of RL greater than 200 Ω. Again, a more comprehensive analysis is necessary to come up with the best harvester configuration for a set of particular requirements imposed. For Model 2 similar requirements as those in Model 1 are established, namely: the power ranges from 150 mW to 600 mW, the primary current equals 5A, the voltage window at the input of the system ranges from 1 V to 3.3 V, the THD should be below 9% and Ns = 200 turns. Table 2 shows the possible harvester configurations.

To harvest more than 150 mW, for the startup primary current, the compensating capacitor should take values between 12 and 14 µF with the corresponding load resistance. The pairs of C_L_ and R_L_ to some extent could be regarded as the matching impedance for the harvester for a certain level of core saturation. 

## 4. Verification of the Model Accuracy. Experimental Results and Energy Harvester Prototype

**Figure 7:**
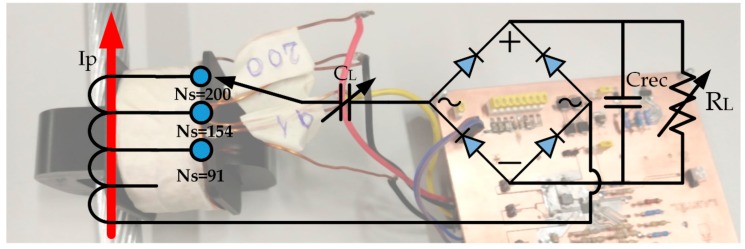
Circuit schematic diagram of the prototype of the harvester.

The accuracy of the models proposed is verified by developing a prototype of the harvester. The simulation results are compared with the measurements obtained by different configurations of the prototype. Figure 7 shows the circuit schematic diagram of the harvester.

The energy harvester consists of a CT with a Si-steel magnetic core characterized by the B-H curve shown in Figure 3. The CT used is of window-type in which the primary winding consists of the high power line passing through the window in a split magnetic core. Therefore, the primary winding is not an integral part of the CT. The prototype harvester has the characteristics listed in Table 3. 

The choice of the material and size of the core relies on several variables. Firstly, the power capability of the core in terms of the amount of power harvested as a function of the primary current i.e., mW per A in I_p_. Secondly, the core window area should provide sufficient space for the primary wire and the secondary winding. Thirdly, the core should have a high saturation flux density, Bsat. The higher Bsat the greater the amount of power that can be extracted since more magnetic energy can be collected by the core. Finally, transmission line operating requirements have to be complied with. A bulky core, for instance, may increase the sag of the transmission line. Two core materials have been considered: ferrite and grain oriented Si-steel material. The final choice of the core material and size was made by using the magnetization curves in the nonlinear inductor in Figure 2 and by simulating them for power comparison purposes. The grain oriented Si-steel was the best option due to the reduction in the size and weight of the magnetic core for the same harvested power. This comparative study has not been included because it is beyond the scope of this paper.

The secondary of the CT has been designed to provide several winding configurations with 91, 154 and 200 turns. Furthermore, a capacitor bank and a potentiometer have been included for resistive load and compensating capacitor definition aiming at simulating different loading conditions. The wire size for the secondary winding is chosen taking into consideration the power losses, which depends on how much current is being drawn from the winding, the length of the wire and the wire resistivity.

**Figure 8:**
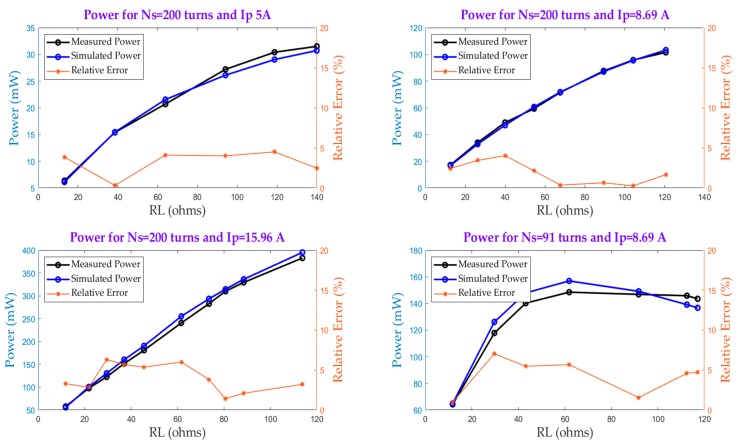
Verification of Model 1 accuracy.

Several harvester configurations have been tested and verified. Regarding the energy harvester based on Model 1, the estimated and measured power along with the relative error for different values of Ns, RL and Ip are evaluated and represented in Figure 8. The power is calculated by measuring the voltage drop and the current flowing across the load resistance. Then, both measured values are multiplied to obtain the harvested power. Under non-saturation conditions, which corresponds with the linear section in the curves in Figure 8, the harvester is ideally operating as a current source developing any arbitrary voltage required to sustain the current into the load. Considering the wire and the rectifier losses, it can be assumed that the power follows a quadratic relationship with respect to the primary current. For increasing values of RL, the core enters into saturation and the power increase is no longer linear on account of the lost current through the magnetization inductance. This fact is more apparent when the number of turns in the secondary is reduced, as can be seen in Figure 8 for Ns = 91 turns. It can also be seen that the maximum relative error of the simulated power is always less than 7%, which confirms the reasonable accuracy of Model 1 for characterization purposes. 

**Figure 9:**
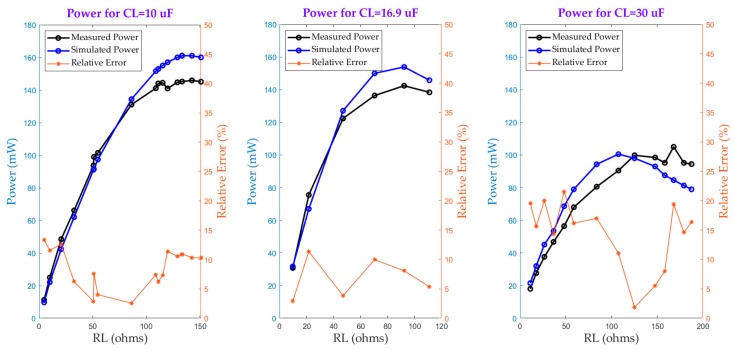
Verification of Model 2 accuracy.

As far as Model 2 is concerned, Figure 9 shows the measured and simulated power as a function of R_L_, C_L_ for N_S_ = 200 and I_p_ = 5 A. 

The power is calculated in the same way as that used in Model 1 and the same conclusions can be reached regarding the influence of the core saturation upon the output power. However, for a startup current of 5 A when reactive power compensation is achieved, the output power obtained is larger than that of Model 1. This power critically depends on the value of the compensating capacitor. For example, for C_L_ = 30 μF, the output power is lower than in the cases when C_L_ = 10 μF and C_L_ = 16.9 μF. This fact was seen in Figure 6, which showed that for values of C_L_ greater than 20 μF the output power sharply decreases. Hence, the output power is very sensitive to the value of C_L_, which depends on the value of the magnetizing inductance, L_m_. The latter exhibits great variability under core saturation in both the knee region and in deeper saturation, which hinders the ability of the model to better estimate the compensating capacitor under such conditions. This is the reason behind the increase in the relative error for C_L_ = 30 μF. According to the relative error range, Model 2 is reasonably precise, allowing the range of potential capacitor values to be estimated for maximum power extraction.

In order to assess the performance of the proposed inductive electromagnetic energy harvester for high voltage power lines, the common standards of power per unit of magnetic core volume, i.e., power density, and power per unit of the primary current can be used. However, for comparison purposes the power density in mW/cm^3^ is more suitable, since most works in the literature utilize this parameter. For the worst case scenario, at the startup current of 5 A, the harvested power density can reach 2.79 mW/cm^3^. Therefore, the harvester proposed in this paper outperforms other approaches to electromagnetic inductive energy harvesters [32,33]. In [37] the power density for a primary current of 60A is equal to 45.96 mW/cm^3^. Finally, in [29] considering a primary current of5 A and for a nanocrystalline core, the power density reaches 7.82 mW/cm^3^ and for a ferrite core 1.97 mW/cm^3^. High permeability of nanocrystalline cores account for their best power density, although at the expense of cost and, most importantly, nanocrystalline cores cannot be easily split because of poor mechanical integrity. 

## 5. Conclusions

In this paper, simulation-based characterization of inductive electromagnetic energy harvesters has been analyzed for two different harvester models. Conflicting design goals, such as maximizing the output power while limiting the output voltage levels and keeping the magnetic core at the onset of saturation, have been simultaneously addressed. Furthermore, an additional insight into the relationship among the different parameters involved in the harvester design process, has been provided. With an eye to potential applications, an experimental estimation technique has been developed, such that the variables influencing the performance of any electromagnetic energy harvester can be evaluated from arbitrary values of output power, primary current, output voltage range and level of core saturation, to name but a few. Two significant contributions have been made in the work presented in this paper: (i) by using a simple MATLAB/Simulink electrical circuit based on a current transformer, an inductive electromagnetic energy harvester can be rapidly deployed; (ii) the level of magnetic core saturation has been estimated and controlled through the evaluation of the total harmonic distortion of the secondary current. The performance of the estimation technique has been experimentally validated by creating a prototype for the harvester, which consists of a Si-steel magnetic core and a power conditioning unit. For the worst case scenario with a startup current of 5A, and for a secondary winding of 200 turns, achieving reactive power compensation, the maximum power extracted by the harvester can be as close as 165 mW, which represents a power density of 2.79 mW/cm^3^. The results obtained confirm that the proposed simulation strategy is accurate in predicting the behavior of the harvester for different operating points and under several loading conditions. 

## Figures and Tables

**Table 1:** Harvester configurations for a particular/specific set of requirements.

I_p_(A)	R_L_(Ω)	V_load_(V)	I_load_(mA)	THD (%)	Power (mW)
12	65	3.21	49.42	6.3	158
12	70	3.44	49.15	5.6	169
13	60	3.24	54.08	6.8	175
13	65	3.49	53.81	5.8	188
14	50	2.95	59.04	6.5	174
14	55	3.23	58.76	5.8	189
15	40	2.56	64.05	6.3	164
15	45	2.86	63.74	6.6	182
15	50	3.17	63.45	6.9	201
15	55	3.47	63.16	5	219

**Table 2:** Harvester configurations for specific requirements.

C_L_(μF)	R_L_(Ω)	V_load_(V)	I_load_(mA)	THD (%)	Power (mW)
12	47	2.66	56.72	7.9	151.25
12	37	2.35	63.76	7.2	150.43
13	39	2.48	63.61	7.5	157.80
13	41	2.60	63.48	7.8	165.22
14	31	2.16	69.76	8.7	150.89

**Table 3:** Harvester parameters.

Parameter	Value	Unit
Core material	Silicon steel	
Magnetic path length (leff)	19.70	cm
Cross-sectional area (Aeff)	312	mm^2^
Core window area	1000	mm^2^
Weight	0.420	Kg
Ns	200, 154, 91	turns
Winding wire diameter	1	mm
Average length per turn	80	mm
Maximum height	25	mm
Saturation magnetic flux density, B_sat_	1.7	T

## References

[1] Weimer M.A., Paing T.S., Zane R.A. Remote area wind energy harvesting for low-power autonomous sensors. Proceedings of the 37th IEEE Power Electronics Specialists Conference.

[2] Orrego S., Kourosh S., Andre R., Kyle D., Brett C., Rajat M., Sung H.K. (2017). Harvesting ambient wind energy with an inverted piezoelectric flag. Appl. Energy.

[3] Perez M., Boisseau S., Reboud J.L. Design and performance of a small-scale wind turbine exploiting an electret-based electrostatic conversion. Proceedings of the Journal of physics: Conference series.

[4] Perez M., Boisseau S., Geisler M., Despesse G., Reboud J.L. A triboelectric wind turbine for small-scale energy harvesting. Proceedings of the Journal of Physics: Conference Series.

[5] Li Y., Shi R. (2015). An intelligent solar energy-harvesting system for wireless sensor networks. EURASIP J. Bioinf. Syst. Biol..

[6] Shaikh F.K., Zeadally S. (2016). Energy harvesting in wireless sensor networks: A comprehensive review. Renewable Sustainable Energy Rev..

[7] Habibzadeh M., Hassanalieragh M., Ishikawa A., Soyata T., Sharma G. (2016). Hybrid solar-wind energy harvesting for embedded applications: Supercapacitor-based system architectures and design tradeoffs. IEEE Circuits Syst. Mag..

[8] Wei C.F., Jing X.J. (2017). A comprehensive review on vibration energy harvesting: Modelling and realization. Renewable Sustainable Energy Rev..

[9] Nasiri A., Zabalawi S.A., Jeutter D.C. (2011). A linear permanent magnet generator for powering implanted electronic devices. IEEE Trans. Power Electron..

[10] Zergoune Z., Kacem N., Bouhaddi N. (2019). On the energy localization in weakly coupled oscillators for electromagnetic vibration energy harvesting. Smart Mater. Struct..

[11] Mahmoudi S., Kacem N., Bouhaddi N. (2014). Enhancement of the performance of a hybrid nonlinear vibration energy harvester based on piezoelectric and electromagnetic transductions. Smart Mater. Struct..

[12] Abed I., Kacem N., Bouhaddi N., Bouazizi M.L. (2016). Multi-modal vibration energy harvesting approach based on nonlinear oscillator arrays under magnetic levitation. Smart Mater. Struct..

[13] Mann B.P., Sims N.D. (2009). Energy harvesting from the nonlinear oscillations of magnetic levitation. J. Sound Vib..

[14] Drezet C., Kacem N., Bouhaddi N. (2018). Design of a nonlinear energy harvester based on high static low dynamic stiffness for low frequency random vibrations. Sens. Actuators, A.

[15] Yang B., Lee C.K., Xiang W.F., Xie J., He J.H., Kotlanka R.K., Low S.P., Ping S. (2009). Electromagnetic energy harvesting from vibrations of multiple frequencies. J. Micromech. Microeng..

[16] Sari I., Balkan T., Kulah H. (2008). An electromagnetic micro power generator for wideband environmental vibrations. Sens. Actuators. A.

[17] Yildiz F., Coogler K.L. (2014). Low power energy harvesting with a thermoelectric generator through an air conditioning condenser. ASEE Annu. Conf. Expostition.

[18] Hyland M., Hunter H., Liu J., Veety E., Vashaee D. (2016). Wearable thermoelectric generators for human body heat harvesting. Appl. Energy.

[19] Lu Z.S., Zhang H.H., Mao C.P., Li C.M. (2016). Silk fabric-based wearable thermoelectric generator for energy harvesting from the human body. Appl. Energy.

[20] Ren J., Hu J., Zhang D.Y., Guo H., Zhang Y.X., Shen X.M. (2018). RF energy harvesting and transfer in cognitive radio sensor networks: Opportunities and challenges. IEEE Commun. Mag..

[21] Mishra D., De S., Jana S., Basagni S., Chowdhury K., Heinzelman W. (2018). Smart RF energy harvesting communications: Challenges and opportunities. IEEE Commun. Mag..

[22] Soyata T., Copeland L., Heinzelman W. (2016). RF energy harvesting for embedded systems: A survey of tradeoffs and methodology. IEEE Commun. Mag..

[23] Chang K.S., Kang S.M., Park K.J., Shin S.H. (2012). Electric field energy harvesting powered wireless sensors for smart grid. J. of Electr. Eng. Technol..

[24] Cetinkaya O., Akan O.B. (2017). Electric-field energy harvesting in wireless networks. IEEE Wirel. Commun..

[25] Yeesparan S., Mohd Z.B.B., Norashidah B.M.D., Mohamad H.H. (2018). A review of energy harvesting methods for power transmission line monitoring sensors. Int. J. of Eng. Technol. (UAE).

[26] Guo F., Hayat H., Wang J. Energy harvesting devices for high voltage transmission line monitoring. Proceedings of the IEEE Power Energ. Soc. Gen. Meeting.

[27] Zhao D.S., Dai D., Li L.C. (2015). Electric field energy harvesting for on-line condition-monitoring device installed on high-voltage transmission tower. Electron. Lett.

[28] Roscoe N.M., Judd M.D. (2013). Harvesting energy from magnetic fields to power condition monitoring sensors. IEEE Sens. J..

[29] Dos S.P.M., Vieira D.A., Rodriguez Y.P.M., Souza C.P., Moraes T.O., Freire R.C. Energy harvesting using magnetic induction considering different core materials. Proceedings of the IEEE International Instrumentation and Measurement Technology Conference (I2MTC) Proceedings.

[30] Hosseinimehr T., Tabesh A. (2016). Magnetic field energy harvesting from AC lines for powering wireless sensor nodes in smart grids. IEEE Trans. Ind. Electron..

[31] Moon J., Leeb S.B. (2014). Analysis model for magnetic energy harvesters. IEEE Trans. Power Electron..

[32] Yuan S., Huang Y., Zhou J.F., Xu Q., Song C.Y., Yuan G.Q. (2016). A high-efficiency helical core for magnetic field energy harvesting. IEEE Trans. Power Electron..

[33] Liu Y.D., Xie X.L., Hu Y., Qian Y., Sheng G.H., Jiang X.C., Liu Y.L. (2016). A novel high-density power energy harvesting methodology for transmission line online monitoring devices. Rev. Sci. Instrum..

[34] Paul S., Chang J.H. (2019). Design of novel electromagnetic energy harvester to power a deicing robot and monitoring sensors for transmission lines. Energy Convers. Manage..

[35] Gupta V., Kandhalu A., Rajkumar R.R. Energy harvesting from electromagnetic energy radiating from AC power lines. Proceedings of the 6th Workshop on Hot Topics in Embedded Networked Sensors.

[36] Ottman G., Hofmann H.F., Bhatt A.C., Lesieutre G.A. (2001). Adaptive piezoelectric energy harvesting circuit for wireless, remote power supply. Proceedings of the 20th AIAA Applied Aerodynamics Conference.

[37] Qian Z.N., Wu J., He X.N., Lin Z.Y. (2018). Power maximised and anti-saturation power conditioning circuit for current transformer harvester on overhead lines. IET Power Electron..

[38] Lefeuvre E., Audigier D., Richard C., Guyomar D. (2007). Buck-boost converter for sensorless power optimization of piezoelectric energy harvester. IEEE Trans. Power Electron..

[39] Kong N.A., Ha D.S., Erturk A., Inman D.J. (2010). Resistive impedance matching circuit for piezoelectric energy harvesting. J. Intell. Mater. Syst. Struct..

[40] Moghe R., Divan D., Deepak, Lambert F. Powering low-cost utility sensors using energy harvesting. Proceedings of the 14th European Conference on Power Electronics and Applications.

[41] LI P., Wen Y.M., Zhang Z.Q., Pan S.Q. (2015). A high-efficiency management circuit using multiwinding upconversion current transformer for power-line energy harvesting. IEEE Trans. Ind. Electron..

[42] Simjee F., Chou P.H. Everlast: long-life, supercapacitor-operated wireless sensor node. Proceedings of the international symposium on Low power electronics and design.

[43] Porcarelli D., Spenza D., Brunelli D., Cammarano A., Petrioli C., Benini L. (2014). Adaptive rectifier driven by power intake predictors for wind energy harvesting sensor networks. IEEE J. Emerging Sel. Top. Circuits Syst..

[44] Jiang X., Polastre J., Culler D. Perpetual environmentally powered sensor networks. Proceedings of the 4th international symposium on Information processing in sensor networks.

[45] MathWorks Documentation. https://es.mathworks.com/help/physmod/sps/examples/nonlinear-inductor-characteristics.html.

